# Characterization of immunomodulatory factors and cells in bronchoalveolar lavage fluid for immune checkpoint inhibitor-related pneumonitis

**DOI:** 10.1007/s00432-023-04696-0

**Published:** 2023-03-21

**Authors:** Peng-Mei Wang, Zhong-Wei Zhang, Shan Zhang, Qian Xing, Zhi-Yong Zhao, Qiong-Hua Lin, Li-Hua Shen, Zhi-Li Xia, Fang-Fang Li, Biao Zhu

**Affiliations:** Department of Critical Care, Fudan University Shanghai Cancer Center, 270 Dong’an Road, Xuhui District, Shanghai, 200032 China

**Keywords:** Checkpoint inhibitors, Immune-related adverse side effects, Bronchoscopy, Pneumonitis, Biologic mechanism, Biomarker

## Abstract

As immune checkpoint inhibitors (ICIs) are widely used, a series of immune-related adverse events (irAEs) have been reported, including immune checkpoint inhibitor-related pneumonitis (ICI-pneumonitis). The incidence of ICI-pneumonitis is higher in reality than in clinical trials. The diagnosis is challenging, mainly based on clinical and imaging features, and requires the exclusion of other causes. The data on the biological mechanisms of ICI-pneumonitis are scarce, resulting in little knowledge of the best treatment for ICI-pneumonitis. Bronchoalveolar lavage (BAL) may be helpful to identify the biological differences or find predictive biomarkers, and may in turn help to develop phenotype-specific targeted drugs to treat ICI-pneumonitis. Herein, we outline the characterization of immunomodulatory factors and cells in bronchoalveolar lavage fluid for ICI-pneumonitis. Through careful sorting and literature review, we find crosstalk between pathogenic Th17/Th1 cells (i.e., Th17.1) and pro-inflammatory monocytes, and activation of Th17(/Th1)/IL-17A (/IFN-γ) pathways may play a key role in the pathogenesis of ICI-pneumonitis. Disruption of the interaction between pathogenic Th17/Th1 cells and pro-inflammatory monocytes (such as, anti-IL-23) may be a potential treatment for ICI-pneumonitis. We first describe the possible pathophysiological mechanisms of ICI-pneumonitis, hoping to contribute to the optimization of diagnosis and treatment, as well as provide readers with research inspiration.

**Supplementary Information:**

The online version contains supplementary material available at 10.1007/s00432-023-04696-0.

## Introduction

With the broad use of ICIs, an increasing incidence of ICI-pneumonitis has been reported. According to clinical trial data, the morbidity of ICI-pneumonitis was about 3–5% (Khunger et al. 2017; Forde et al. 2018), which was previously considered uncommon. However, in the real world, ICI-pneumoniti is reported more frequently (Cho et al. 2018; Suresh et al. 2018). With ICI monotherapy for nonsmall cell lung cancer (NSCLC), ICI-pneumonitis rates range from 1 to 12% at all grades (Nishino et al. 2016a), a grading of immune-related adverse events based on the National Cancer Institute Common Terminology Criteria for Adverse Events (CTCAE) Version 5.0 (US Department of Health and Human Services 2017) (Supplemental Table1). ICI-pneumonitis is one of the most common fatal irAE (Nishino et al. 2016b; Delaunay et al. 2017). The diagnosis of ICI-pneumonitis is difficult because it has no specific diagnostic biomarkers. The diagnosis mostly depends on clinical symptoms and high-resolution CT (HRCT) of the chest, which is not specific. The Society for Immunotherapy of Cancer (SITC) and American Society of Clinical Oncology (ASCO) suggest BAL and/or TBLB for doubtful ICI-pneumonitis of grade 2 and above (Puzanov et al. 2017a; Brahmer et al. 2018). According to American Thoracic Society (AST) clinical practice guideline, BAL site should be based on HRCT, with a minimal volume of 5 ml of BAL sample (Meyer et al. 2012). Whereas this section has not been well described in previous papers, this paper aims to describe the changes of immunomodulatory factors and cells in bronchoalveolar lavage fluid in the hope of finding potential diagnostic biomarkers or therapeutic targets.

## BAL lymphocytosis may be an adjunct to diagnosis ICI-pneumonitis in certain clinical cases

A cell pattern analysis of BAL can be useful in diagnosing interstitial lung diseases (Meyer et al. 2012). Recently, more and more data suggest that BAL cell analysis can offer diagnostic cues to ICI-pneumonitis by uncovering the popularity of lymphocytosis. Strippoli et al. (2020) conducted a BAL analysis of 5 patients with metastatic melanoma who suffered from ICI-pneumonitis. All cases showed increased CD8^+^ T cells, reversed CD4^+^/CD8^+^ ratios, and neutrophil, eosinophil, natural killer cell, and B cell ratios within normal limits. A similar study also found that the percentage of lymphocytes increased by more than 20% in all ICI-pneumonitis patients (Nishiyama et al. 2021). Delaunay et al. (2017) reported BAL findings in 35 samples from a cohort of 64 ICI-pneumonitis patients, and 68.5% of these patients have BAL lymphocytes > 15%. However, in a study by (Sheshadri et al. 2022), lymphocytosis was not a distinctive feature of pneumonitis in patients with leukemia who underwent BAL. In addition, eosinophils were also found in some cases (Hara et al. 2021). Nevertheless, BAL lymphocytosis may be an adjunct to diagnosis ICI-pneumonitis in certain clinical cases, and larger studies are needed. Anyway, these studies offer a clear diagnostic tool and indicate that differential BAL cell counts can be used as a component of the clinical assessment protocol for patients with suspected ICI-pneumonitis. Alternatively, BAL is also used to rule out infection and cancer etiology. BAL cell analysis combined with computed tomography scan patterns can better define and separate ICI-pneumonitis from other types of interstitial lung disease (Strippoli et al. 2020).

## Analysis of cytokines and immune cells in BALF helps to understand the potential mechanisms of ICI-pneumonitis

ICI-pneumonitis is disseminated and comparatively uncommon in single hospitals, making systematic studies difficult. The paucity of articles on the biological mechanisms of ICI-pneumonitis has led to little knowledge of the best treatment for ICI-pneumonitis. Moreover, it is uncertain whether there is a connection between the potential biological mechanism and the specific clinical and imaging presentation. Multidisciplinary collaboration, in particular the provision of clinical research specimens (blood, BALF, and lung pathology samples), may be helpful to identify the biological differences and in turn help to develop phenotype-specific targeted drugs to cure or prevent ICI-pneumonitis (Sears et al. 2020). Here we summarize the biological and immunologic characteristics of ICI-pneumonitis in BALF by collecting recent data and trying to elucidate the possible physiological mechanisms.

## BALF cytokines changes in ICI-pneumonitis patients

Suresh et al. (2019) observed lower levels of neutrophil chemoattractant IL-8 and macrophage-inflammatory protein-3α (MIP-3α or CCL20), in patients with ICI-pneumonitis BALF. Inflammatory and infectious diseases have been linked to MIP-3. The decrease in MIP-3α, together with reduced IL-8 levels and paucity of neutrophil dominance in BALF further boost the idea that ICI-pneumonitis is not a phenomenon triggered by bacterial infection. Furthermore, they found elevated levels of IL-12p40, significantly higher levels of IFN-γ-induced protein 10 (IP-10, also known as CXCL-10), and the levels of the T-cell chemotactic protein TARC (also named CCL17) were on the rise. They also found that increased CCL17 levels correlated with increased CD11b^hi^ populations of myeloid cells. CCL17 is a chemokine of Th17. CD11b^+^ cells are an important source of CCL17 chemokines in the lung (Medoff et al. 2009). IL-12 is the coordinator of type I polarization. IP-10 is a member of the CXC family of cytokines and can be induced by IFN-γ. Its receptor CXCR3 is highly expressed on activated Th1 cells. Thus, it can recruit Th1 cells at sites of inflammation. In a Th1 mouse model of lung injury, IP-10 was significantly increased in the inflammatory focal lung as a direct consequence of IFN-γ production by activated Th1 cells. This, in turn, leads to an amplification of the Th1-mediated-inflammatory response, resulting in a positive feedback loop (Dixon et al. 2000). In addition, IP-10 (CXCL-10) can guide Tcm lymphocytes to their destination within lymph nodes. These BAL cytokine data suggest that type 1 and type 17 polarization are involved in the pathobiology of ICI-pneumonitis.

Intriguingly, the effector cytokines IL-17A and IFN-γ of Th17 and Th1 cells were also found to be elevated in ICI-pneumonitis patients. In the study by Kowalski et al. (2022), the expression of IL-6 in BALF was remarkably higher in ICI-pneumonitis patients compared to all control groups. Similarly, the level of IL-17A was significantly elevated as compared to lung cancer and interstitial lung disease (ILD). In addition, there was a significant overall increase of IFN-γ. However, they didn’t find particularly increased cytokines in serum. These findings were consistent with the work by (Kim et al. 2021), where IL-6, IL-17A and IFN-γ levels in BALF were higher in the ICI-pneumonitis group than in the control group. Nonetheless, perhaps because the sample was too small, there was no statistical significance. In addition, both BALF and peripheral blood levels of IL-17A and IL-35 were significantly higher at the time of ICI-pneumonitis diagnosis compared to baseline levels in the study by (Wang et al. 2020). They also found that IL-17A was not only correlated with the development of ICI-pneumonitis but also was a useful predictor of the severity of ICI-pneumonitis. Notably, IL-6, IL-17A and IFN-γ were not found to be elevated in the study by (Suresh et al. 2019). This may be related to differences in disease severity, sampling time, changes in cytokine dynamics and experimental platform.

Both IFN-γ, IL-6, and IL-17A are important proinflammatory cytokines. Traditionally, IFN-γ is regarded as the hallmark proinflammatory cytokine for Th1-dominated autoimmune processes. It can strongly enhance macrophage activation and improves antigen presentation by antigen-presenting cells (APCs) (Zhang et al. 2019). Remarkably, IFN-γ inhibited Th17-mediated autoimmunity in murine (Kelchtermans et al. 2008), and also inhibited fibrosis in a variety of models, including viral hepatitis, bleomycin-induced pulmonary fibrosis and schistosomiasis-induced fibrosis (Wynn 2004). Growing evidences show that IFN-γ exerts both promotive and inhibitory roles in autoimmune diseases via Jak-STAT pathway, which depends on the type and duration of the disease, the site and intensity of IFN-γ action. IFN-γ is an essential mediator of immunity and inflammation, and possesses a crucial homeostatic function (Hu and Ivashkiv 2009).

IL-17 is a Th17 cytokine that has been associated with the pathogenesis of many autoimmune diseases. IL-17A acts on a variety of cells, including airway epithelial cells, airway smooth muscle (ASM) and lung microvascular endothelial cells (LMVECs) (Tsai et al. 2013). By activating IL-17R/NF-κB signaling, airway epithelial cell proliferation is enhanced and epithelial−mesenchymal transition (EMT) of alveolar epithelial cells is directly induced. In a bleomycin-induced lung fibrosis model, IL-17 remarkably boosted lung fibroblast proliferation and enhanced smooth muscle actin, type I and III collagen expression in fibroblasts (Dong et al. 2013). Both inhibition of IL-17RA by siRNA and suppression of NF-κB silenced the fibroblast response to IL-17RA stimulation. These results suggest a direct effect of the IL-17A/IL-17RA axis in facilitating human tissue remodeling through direct action on lung fibroblasts (Zhang et al. 2019).

IL-17A may cause human airway epithelial cells to be insensitivity to corticosteroids. In human fibroblast MRC5 cells, IL-17 increased type I collagen production, upregulated glucocorticoid receptor β (GR-β) expression, and impaired the inhibitory effect of glucocorticoids on type I collagen and extracellular matrix production in pulmonary fibrosis (Lo et al. 2022). A recent study showed that IL-17A does inhibited typical corticosteroid genes, such as HSD11B2 and FKBP5 (Rahmawati et al. 2022). These findings suggest that IL-17A may be associated with corticosteroid insensitivity in interstitial lung disease.

IL-6 is also a proinflammatory cytokine. It can induces enhanced T-cell proliferation and Th 17-cell polarization. Thus, increased IL-6 would further stimulate Th17 cells to produce IL-17A. IL-6 is rarely useful for a single diagnostic tool, due to its broad inflammatory response. However, IL-6 in BALF appears to play a relevant role in understanding of the pathophysiology of ICI-pneumonitis, which may be a targetable cytokine for therapy. Table 1 summarizes the key cytokines and immune cells in BALF of patients with ICI-pneumonitis.

**Table 1:** Summary of key cytokines and immune cells in BALF of patients with  ICI-pneumonitis

Changes of cytokines in BALF		The landscape of immune cells in BALF	
References	Cytokines	References	Cells
Suresh et al. (2019)	IL-8↓	Suresh et al. (2019)	Tcms,CD4^+^CD45RA^−^CD62L^+^ ↑
	MIP-3α↓		TNF-α^hi^ IFN-γ^+^ Th1 ↑
	IL-12p40↑		PD-1^hi^/CTLA-4^hi^ CD4^+^ Tregs↓
	IP-10/CXCL10↑		Tems, TNF-α^hi^ IFN-γ^+^ CD8^+^ ↑
	TARC/CCL17↑		IL-1β^hi^ TNF-α^hi^ CD11b^hi^ monocytes ↑
Kowalski et al. (2022)	IL-6↑	Kim et al. (2021)	T-bet^+^ RORγt^+^ (Th1) ↑
Kim et al. (2021)	IL-17A↑		CXCR3^+^ T-bet^+^ CCR6^+^ RORγt^+^ IFN-γ^+^ IL-17^+^ Th17/Th1 (i.e., Th17.1) ↑
	IFN-γ↑		Tems,CD45RA^−^ CCR7^−^ IFN-γ^+^ CD8^+^ ↑
Wang et al. (2020)	IL-17A↑		/
	IL-35↑		
	/	Franken et al. (2022)	T-bet^+^ ROR-γ^+^ IFN-γ^+^ IL-17A^+^ GM-CSF^+^ CD4^+^ T_H17.1_ ↑
			CD8^+^ T_EM_ ↑
			IL-1β^hi^ monocytes ↑

### The alveolar immune cell landscape in ICI-pneumonitis

To further understand the biological mechanisms of ICI-pneumonitis, (Suresh et al. 2019) analyzed the BAL immune cell population by flow cytometry and identified several immune cell subsets that may play pivotal roles in the development of ICI-pneumonitis. First, a significantly higher proportion of central memory CD4^+^ T cells (Tcms, CD4^+^CD45RA^−^CD62L^+^) were observed in the ICI-pneumonitis samples. When compared with other conventional T cells, Tcms are more tolerant of steroid-induced apoptosis. This may help to explain why up to 40% of patients with ICI-pneumonitis fail high-dose steroid therapy (Suresh et al. 2019, 2018). Secondly, Th1-cell subsets were upregulated in ICI pneumonitis, producing large amounts of IFN-γ and TNF-α. Th1 cells are known to mediate cellular immunity, whose signature cytokine is IFN-γ (Kaiko et al. 2007). It is associated with various lung diseases, including pulmonary sarcoidosis, and hypersensitivity pneumonitis (Prasse et al. 2000). The enhanced Th1 response during the development of ICI-pneumonitis implies that Th1 polarization may provoke an overactive T-cell response in ICI-pneumonitis patients. Thirdly, the number of PD-1^hi^/CTLA-4^hi^ CD4^+^ Tregs (i.e. CD4^+^ FoxP3^+^ Tregs) was reduced, implying a diminished Treg suppressive phenotype. The reduction of Treg suppression may promote lively Th1-cell immune responses, and contributes to unchecked immune dysregulation seen in ICI-pneumonitis. Also, IL-1β^hi^TNF-α^hi^CD-11b^hi^ monocytes were dramatically upregulated in ICI-pneumonitis. Interestingly, they did not observed an increase in soluble IL-1β and TNF-α. Possible explanation is that the inflammation promotes the translation and endosomal storage of IL-1β, but not its release from the membrane. Another possibility is that the release of IL-1β occurs earlier in the injury and decreases over time as samples are collected (Suresh et al. 2019). As above mentioned, CD11b^+^ cells are a major source of CCL17 chemokines. Thus, IL-1β^hi^TNF-α^hi^CD-11b^hi^ monocytes may promote type 17 polarization. Finally, there was an elevation of TNF-α^hi^ IFN-γ^hi^ CD8^+^ cells in ICI-pneumonitis. By producing increased levels of IFN-γ and TNF-α in vivo, they can boost type 1 immune responses. Activated helper CD4^+^ T cells can recruit effector CD8^+^ T cells to peripheral tissues through attracting release CXCL9 and CXCL10 (Nakanishi et al. 2009). Meanwhile, several studies have shown that inflammatory cytokines IL-12 and type I IFNs can drive the expansion of CD8^+^ T cell and the formation of effector and memory cell (Arens and Schoenberger 2010).

Kim et al. (2021) studied patients with leukemia who underwent aggressive pretreatment. They classified CD4^+^ T cell subsets into Th1 (Tbet), Th2 (GATA3), Th17 (RoRγT), and Treg (FoxP3) based on chemokine/cytokine receptor and key transcription factor expression. They observed that Th1 and Th17/Th1 (i.e., Th17.1 cells) cells were enriched in ICI group. Next, they evaluated the functionality of T cells in BALF. The results showed that the absolute densities of IFN-γ-producing and/or IL-17-producing T cells were greater in ICI-pneumonitis patients than in controls, especially IFN-γ^+^ IL-17^+^ CD4^+^ T cells (i.e. functional definition of Th17.1 cells). Th17.1 cells are a subset of Th17 lineage, but have the traits of IL-17/IFN-γ double producing. They have increasingly been shown to act as pathogenic drivers in autoimmune processes such as Crohn’s disease, multiple sclerosis, rheumatoid arthritis and sarcoidosis (Stadhouders et al. 2018). Furthermore, Th17.1 cells have been shown to upregulate inflammation and be resistant to glucocorticoids. On the other hand, the frequency of BAL CD8^+^ T cells was dramatically increased in CIP patients as compared to the control group. These cells were predominantly CD45RA^−^ CCR7^−^ IFN-γ^+^ CD8^+^ effector memory cells, similar to the findings of (Suresh et al. 2019).

They also characterized lymphocytes in BALF and peripheral blood (PB) from the ICI group and controls. They found that lymphocyte frequency and T cell clonality were higher in the ICI group as compared to controls in both BAL and peripheral blood (PB). Wang et al. (2020) measured the concentration of IL-17A in peripheral blood and BALF. Meanwhile, the centages of Th1, Th2, and Th17 cells, and Tregs were synchronically tested in peripheral blood. Higher numbers of Th1 and Th17 cells, and higher Th17/Tregs and Th1/Th2 cell ratios, were observed during ICI-pneumonitis development compared to baseline. Serum IL-17A levels were strongly associated with Th1, Th17 cell percentage, and Th17/Tregs ratio. These results suggested that ICI-pneumonitis might be a systemic inflammation.

Franken et al. (2022) performed single-cell transcriptomics on BALF of 11 patients with ICI-pneumonitis in a prospective observational study. They observed extensive accumulations of T cells in ICI-pneumonitis BALF, mainly pathogenic Th17.1 cells (i.e., Th17/Th1 cells). These cells accounted for one third of all T cells, and simultaneously expressed TBX21 (encoding T-bet) and RORC (ROR-γ), IFN-G (IFN-γ), IL-17A, CSF2 (GM-CSF). Besides, they also analyzed innate immune cells, and found a significant increase of pro-inflammatory IL-1β^high^ monocytes, which expressed high levels of pro-inflammatory genes, including TNF (encoding TNF-α), IL-6, IL-23A, GM-CSF receptor, CCL20 and the interferon- induced chemokines CXCL9/10. The receptors for CXCL9/10 and CCL20 are CXCR3 and CCR6, respectively, which are both expressed in Th17.1 cells (Metzemaekers et al. 2018 Jan; Schutyser et al. 2003). Thus, IL-1β^high^ monocytes may recruit Th17.1 cells by expressing CXCL9/10 and CCL20, which target CXCR3 and CCR6 in ICI-pneumonitis. Also, monocyte-derived IL-23 and IL-1β upregulate GM-CSF expression in Th17.1 cells (El-Behi et al. 2011). Th17.1 cell-derived GM-CSF then reversely targets GM-CSF receptor monocytes and leads to expanded inflammation and damage to lung tissue. This culminates in a damaging feedback loop. CD8^+^ cells were also more abundant in ICI-pneumonitis BALF compared to controls, mainly effector memory T cells.

**Fig. 1:**
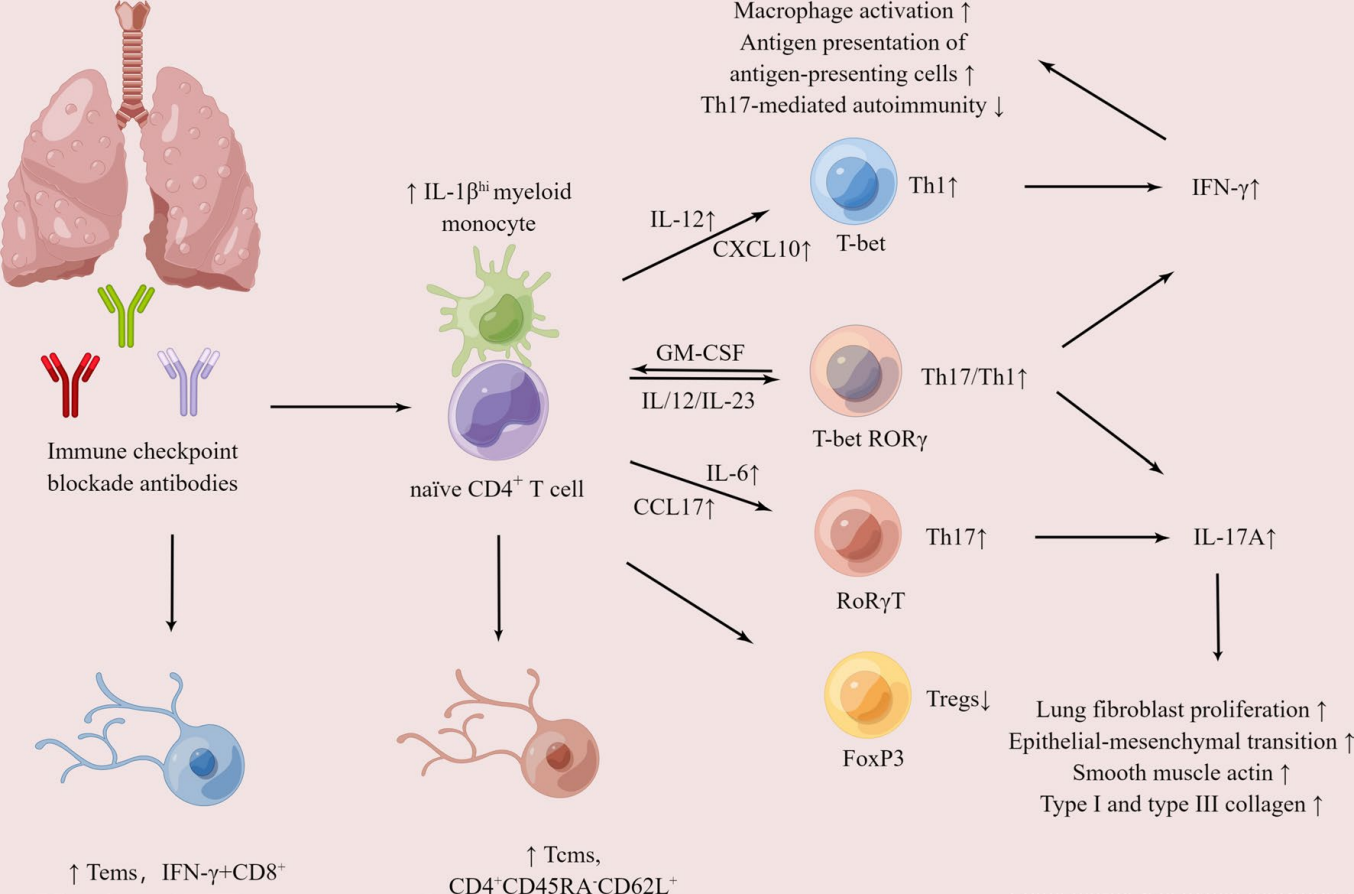
Characterization of immunomodulatory factors and cells in BALF for immune checkpoint inhibitor-related pneumonitis. Immune checkpoint inhibitors destroy immune tolerance and enhance T-cell activation and immune response. Overactivated T cells interact with pro-inflammatory monocytes to increase the release of chemokines IL-12p40, CXCL9/10, CCL17, IL-6, and IL-23A, which promotes the polarization of naive CD4^+^ cells to pro-inflammatory Th1, Th17(/Th1) cells, meanwhile the decrease of anti-inflammatory cells Treg. As a result, the production of effector cytokines IL-6, IFN-γ, and IL-17A is significantly increased, which auto-amplify Th1 and Th17 responses and achieve the positive feedback loop, ultimately leading to lung tissue damage. In addition, by attractively releasing CXCL9 and CXCL10, activated helper CD4^+^ T cells can recruit effector CD8^+^ T cells to lung tissues

Overall, based on the above studies we speculate that when immune checkpoint inhibitors reactivate T cells to exert antitumor effects, T cells also occur with off-target effects. Overactivated T cells, especially Th17/Th1 cells, promote monocyte to expand inflammation and release chemokines IL-12p40, CXCL9/10, CCL17 and cytokines IL-6, IL-23A, TNF-α by secreting GM-CSF. These immunomodulatory factors further promote the polarization of naive CD4^+^ cells into pro-inflammatory Th1, Th17 or Th17/Th1 cells, meanwhile the decrease of anti-inflammatory cells Treg. As a result, their effector cytokines IFN-γ and IL-17A are significantly increased. IL-17A may promote lung fibroblast proliferation by stimulating IL-17RA, and cause corticosteroid insensitivity in interstitial lung disease via upregulating glucocorticoid receptor β (GR-β) expression or inhibiting typical corticosteroid genes. IFN-γ may exert promotive or inhibitory effects depending on the duration and severity of the disease. In addition, IFN-γ and IL-17A can automatically amplify Th1 and Th17 responses, thus achieving a positive feedback loop that maintains the autoimmune process and ultimately leads to immune checkpoint inhibitor-associated pneumonitis. Moreover, by attractively releasing CXCL9 and CXCL10, activated helper CD4^+^ T cells can recruit effector CD8^+^ T cells to peripheral tissues (Fig. 1).

To date, the treatment for ICI-pneumonitis is mainly corticosteroids. Several guidelines (Brahmer et al. 2018; Puzanov et al. 2017; Haanen et al. 2022) recommend immunomodulatory agents, such as TNF-α inhibitors, anti-IL-6, and intravenous immunoglobulin (IVIG), as second-line treatment for steroid-refractory ICI-pneumonitis. However, these guidelines recommend a similar approach for steroid-refractory irAEs, lacking immunohistopathological etiological classification. Emerging evidence shows that crosstalk between pathogenic Th17/Th1 cells and pro-inflammatory monocytes plays a key role in ICI-pneumonitis. Although anti-TNF-α and anti-IL-6 are successful in some cases, they do not destroy the interaction between Th17/Th1 cells and monocytes (Franken et al. 2022; Stroud et al. 2019). Monocyte-derived IL-12 and IL-23 are activators of Th1 and Th17 polarizaion, respectively. They act as a bridge between the innate and adaptive immune responses. Accordingly, anti-IL12/IL-23 (ustekinumab, which targets both Th1 and Th17(/Th1) responses) and anti-IL-23 (guselkumab, which targets Th17(/Th1) responses) may be attractive options for steroid-refractory ICI-pneumonitis, which have demonstrated clinical efficacy in systemic lupus erythematosus (SLE) and sjögren’s syndrome (SS) (Schinocca et al. 2021).

## Concluding remarks

Due to the lack of specific biomarkers, the diagnosis of ICI-pneumonitis is difficult. Available data suggest that crosstalk between pathogenic Th17/Th1 cells and pro-inflammatory monocytes plays a key role in the development of ICI pneumonitis. Blockage of IL-23/IL-17 axis (such as, anti-IL-12/IL-23, or anti-IL-23) may be potential therapy for ICI-pneumonitis, especially steroid-refractory ICI-pneumonitis. Furthermore, understanding these immunomodulatory factors and cells in BAL may help to identify predictive and diagnostic biomarkers of ICI-pneumonitis. So far, we have not found such articles demonstrating the predictive or therapeutic value of Th17(/Th1) or Th1 cytokines in ICI-pneumonitis. More randomized controlled trials are needed to prove it in the future.

## Supplementary Information

Below is the link to the electronic supplementary material.

Supplementary file1 (DOCX 11 KB)

## Data Availability

We look forward to more randomized controlled trial in the future. Maybe there are available data in the furture.
