# Supplementary material for: Characterization of immunomodulatory factors and cells in bronchoalveolar lavage fluid for immune checkpoint inhibitor-related pneumonitis
Source: J Cancer Res Clin Oncol. 2023 Mar 21;149(10):8019–26. doi: 10.1007/s00432-023-04696-0 (PMC10374683; doi:10.1007/s00432-023-04696-0)
Supplement: Supplementary file 1 — Supplementary file1 (DOCX 11 KB) [file 432_2023_4696_MOESM1_ESM.docx]

**Supplemental Table 1**

**Grading Syststerm of Immune Checkpoin Inhibitors-related Pneumanitis Based on The National Cancer Institute Common Terminology Criteria for Adverse Events(CTCAE) Version 5.0**

| Grade | Symptom |
| --- | --- |
| 1 | Symptomatic; medical intervention indicated; limiting instrumental ADL^✷^ |
| 2 | Symptomatic; medical intervention indicated; limiting instrumental ADL^✷✷^ |
| 3 | Severe symptoms; limiting self care ADL; oxygen indicated |
| 4 | Life-threatening respiratory compromise; urgent intervention indicated (e.g. , tracheotomy or intubation) |
| 5 | Death |

ADL: activities of daily living

*Instrumental ADL refer to preparing meals, shopping for groceries or clothes, using the telephone, managing money, etc.

**Self care ADL refer to bathing, dressing and undressing, feeding self, using the toilet, taking medications, and not bedridden.
